# Synthesis, Immobilization and Catalytic Activity of a Copper(II) Complex with a Chiral Bis(oxazoline)

**DOI:** 10.3390/molecules190811988

**Published:** 2014-08-11

**Authors:** Liliana Carneiro, Ana R. Silva, Peter S. Shuttleworth, Vitaly Budarin, James H. Clark

**Affiliations:** 1 Department of Chemistry, CICECO, University of Aveiro, Aveiro 3810-193, Portugal; 2 Departamento de Física de Polímeros, Elastámeros y Applicaciones Energéticas, Instituto de Ciencia e Tecnología de Polímeros, CSIC, Madrid 28006, Spain; 3 Green Chemistry Centre of Excellence, Department of Chemistry, University of York, York YO10 5DD, UK

**Keywords:** copper(II), bis(oxazoline), homogeneous catalysis, heterogeneous catalysis, asymmetric catalysis

## Abstract

A chiral bis(oxazoline) bearing CH_2_OH groups was synthesized from a commercial bis(oxazoline) and characterized by ^1^H- and ^13^C-NMR, high resolution ESI-mass spectrometry and FTIR. The corresponding copper(II) complex was immobilized onto the surface of a mesoporous carbonaceous material (Starbon^®^ 700) in which the double bonds had been activated via conventional bromination. The materials were characterized by elemental analysis, ICP-OES, XPS, thermogravimetry and nitrogen adsorption at 77 K. The new copper(II) bis(oxazoline) was tested both in the homogeneous phase and once immobilized onto a carbonaceous support for the kinetic resolution of hydrobenzoin. Both were active, enantioselective and selective in the mono-benzoylation of hydrobenzoin, but better enantioselectivities were obtained in the homogeneous phase. The heterogeneous catalyst could be separated from the reaction media at the end of the reaction and reused in another catalytic cycle, but with loss of product yield and enantioselectivity.

## 1. Introduction

Bis(oxazoline) ligands are chiral, privileged ligands that when coordinated to for example copper act as very efficient and enantioselective homogeneous catalysts in several organic transformations, such as cyclopropanation of alkenes, aziridination of alkenes, Diels-Alder reactions, *etc.* [1]. A decade ago they were also found to be homogeneous catalysts in the kinetic resolution of 1,2-diols [2]. Despite their efficiency, selectivity and enantioselectivity, the work-up of the homogeneous phase reaction is cumbersome, since homogeneous catalysts are not easily separated from the products. Although some chiral bis(oxazoline) ligands are commercially available, they are expensive, hindering their industrial applicability [2].

On the other hand, heterogeneous catalysts can be easily separated from the reaction media by simple filtration and then the products can be isolated, often without metal contamination. The recovered heterogeneous catalyst can also be used in further catalytic cycles improving the cost of the process. Therefore, the heterogenization/immobilization of homogeneous catalysts using solid porous supports has been a popular strategy to combine the advantages of both homogeneous and heterogeneous catalysts [3,4,5,6]. Several immobilization strategies have been used; however, covalent attachment of the homogeneous catalysts to the surface of the material increases the resistance of the catalyst to leaching [3,4,5,6]. Due to their availability and low cost, organic polymers have been widely explored as supports for homogeneous catalysts and in particular for copper bis(oxazoline) ligands [3,4,5,6]. Nevertheless, their major drawback has been the lack of porosity and stability upon reuse, especially when using chlorinated solvents [3,4]. With the discovery of MCM-41 and later other materials, such as, SBA-15, ordered mesoporous silicas have become popular supports for the immobilization of homogeneous catalysts [3,4,5,6,7,8,9,10,11,12]. Some success has been achieved using this type of material as support [3,4,5,6,7,8,9,10], especially using mesocelullar silica foams in the case of the copper bis(oxazolines) [11,12]. Nevertheless the immobilization strategies are limited to organosilane reagents, which in itself leads to some issues regarding the remaining free silanols requiring extra silylanation [11,12] or the use of other less acidic mesoporous silicas [8,9,10].

Porous carbonaceous materials are not as explored, despite the wide use of activated carbons as supports in several commercially available heterogeneous catalysts as surfaces rich in oxygen surface groups [13] that allow the design of a wider range of immobilization strategies [14,15,16,17]. The structure of activated carbons is not ordered and is mainly microporous [13]. In 1999, Ryoo *et. al.*, described the synthesis of an ordered mesoporous carbon prepared via nanocasting using MCM-48 as a hard template [18]. This material, as well as other ordered mesoporous carbons prepared in a one-pot synthesis together with the silica precursor, were used as supports for the immobilization of aza-bis(oxazoline) and commercial bis(oxazoline) via organosilane reagents [8,9,10]. These heterogeneous catalysts were active and enantioselective in several organic transformations, but with inferior performance compared to the corresponding ordered mesoporous silicas supports [8,9,10].

**Scheme 1:**
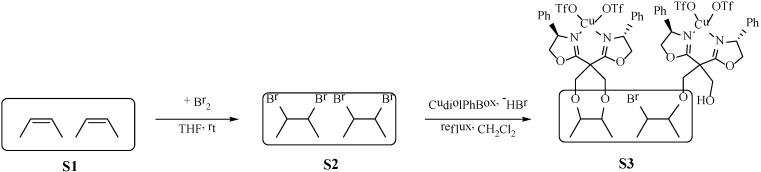
Modification and immobilization strategy of the CudiolPhBox complex (S3) onto the brominated (S2) Starbon ^®^ 700 (S1).

Starbons^®^ are mesoporous carbonaceous materials prepared by the controlled carbonization of expanded starch [19] and other polysaccharides. When carbonized at high temperatures they present a more graphitized surface than the materials carbonized at lower temperatures [19]. These types of materials have never been explored as supports for asymmetric homogeneous catalysts. Herein we report on the immobilization of a copper(II) complex with a commercial bis(oxazoline), functionalized with hydroxyl groups that allowed the covalent attachment to the brominated surface of a Starbon^®^ carbonized to 700 °C (Scheme 1). The material was subsequently applied as a heterogeneous catalyst in the kinetic resolution of a 1,2-diol.

## 2. Results and Discussion

**Scheme 2:**
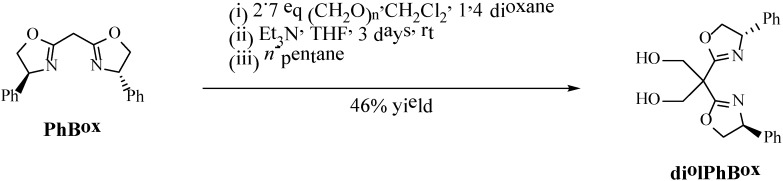
Functionalization of the PhBox ligand with CH_2_OH groups (diolPhBox).

The copper(II) complex with the PhBox bis(oxazoline) was anchored onto the surface of the porous carbonaceous material, Starbon^®^ 700, in a three step procedure according to Scheme 1. Initially, to be able to anchor the ligand onto the Starbon^®^ 700 carbonaceous material, the central carbon of the PhBox ligand bridge was functionalized with CH_2_OH groups (Scheme 2), by adapting procedures described in the literature [20]. To the best of our knowledge this is a new organic molecule and thus it was conveniently and completely characterized by ^1^H- and ^13^C-NMR, high resolution ESI-mass spectrometry and FTIR (see Experimental). This molecule was immobilized onto the surface of the carbonaceous material Starbon^®^ 700 (S1), in which the double bonds had been brominated in order to make them more reactive with the hydroxyl groups [17,21].

### 2.1. Characterization of the Materials

Table 1 shows the elemental analysis of the starting (S1) and chemically modified materials (S2 and S3). As expected, the Starbon^®^ 700 (S1), obtained by the controlled carbonization of starch at 700 °C, has a high content of carbon (91%) like other conventional porous carbonaceous materials, such as activated carbon [13]. The XPS analysis of this material only shows the presence at the surface of the material of carbon in high atomic percentage and oxygen (Table 2). The original Starbon^®^ 700 material possesses both high pore volume and a Brunauer, Emmett and Teller (BET) surface area of 508 m^2^/g, with significant micropore surface impact (Table 3).

**Table 1:** Elemental analysis for the starting Starbon^®^ 700 (S1), brominated (S2) and with the CudiolPhBox complex anchored (S3).

Sample	%C ^a^	%N ^a^	%H ^a^	%S ^a^	%Cu ^b^
S1	90.90	0.41	1.11		
S2	80.05	0.32	1.24		
S3	84.02	0.36	1.12	0.46	0.53

Notes: **^a^** Obtained by elemental analysis; **^b^** Obtained by ICP-OES.

**Table 2:** XPS analysis for the starting Starbon^®^ 700 (S1), brominated (S2) and with the CudiolPhBox complex anchored (S3). **^a^**

Sample	%C	%O	%Br	%N	%F	%S	%Cu
S1	87.65	12.35					
S2	85.62	13.78	0.60				
S3	88.39	9.98	0.32	0.25	0.81	0.19	0.05

Notes: **^a^** Atomic percentage.

**Table 3:** Textural properties for the S1, S2 and S3 samples.

Sample	*A*_BET_ (m^2^/g)^a^	*A*_micro_ (m^2^/g) ^a^	*A*_external_ (m^2^/g) ^a^	V_BJH_^ads^ (cm^3^/g)	D_BJH_^ads^ (nm) ^b^
S1	508	296	212	0.654	10.7
S2	266	86	179	0.571	11.1
S3	203	38	165	0.508	12.1

Notes: **^a^** Obtained by t-plot method; **^b^** Average pore diameter.

**Figure 1:**
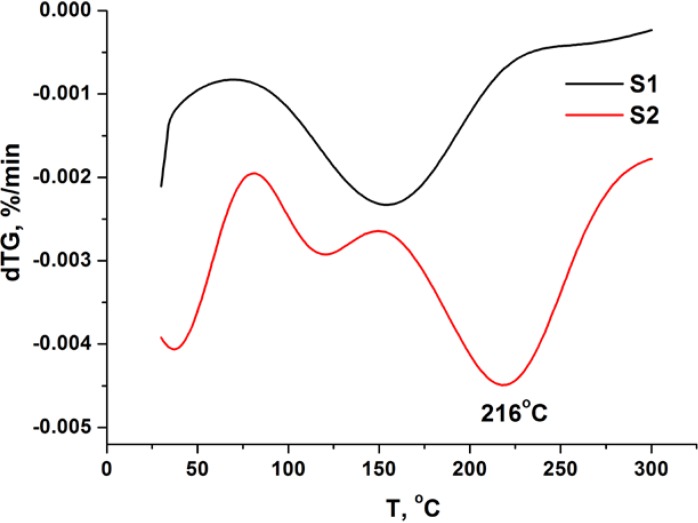
Derivative of thermogravimetric curves for samples S1 and S2.

**Figure 2:**
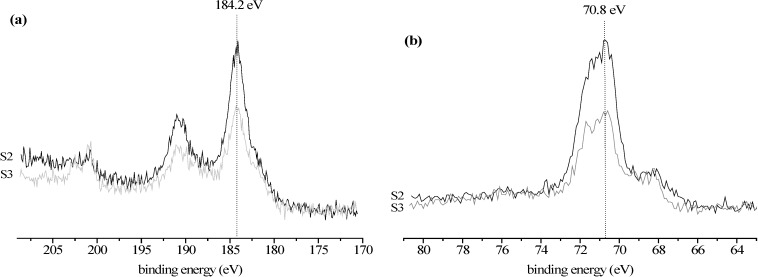
High resolution XPS spectra for the S2 and S3 samples in the regions: (**a**) Br 3p and (**b**) Br 3d.

The surface of Starbon^®^ 700 that is rich in double bonds was then activated with bromine, better leaving group when reacted with hydroxyl groups (Scheme 1) [17,21]. Elemental analysis shows that there is a drastic decrease in the S2 carbon content (more than 10%), showing that bromine was introduced at the surface of the S1 material. This decrease in carbon content agrees well with the first weight loss observed during the thermogravimetric experiments with S2, verifying the presence of bromine (Scheme 1). The derivative of this thermogravimetric curve shows a peak centered around 215 °C (Figure 1). This is corroborated by the XPS analysis showing the presence of bromine corresponding to 463 µmol/g (Table 2). The binding energy of the Br 3p_3/2_ and Br 3d_5/2_ peaks are 184.2 (Figure 2a) and 70.8 eV (Figure 2b), respectively, which are consistent with reported values for Br atoms covalently bonded to carbon atoms [21,22]. Nevertheless, the presence of a smaller peak due to bromide anions can be detected in the XPS spectra (Figure 2) at 182.5 and 68.5 eV, respectively, due to Br 3p_3/2_ and Br 3d_5/2_ peaks, probably due to some hydrolysis of the C-Br bonds [22]. Thus it may be concluded that the bromination of the Starbon^®^ 700 double bonds was achieved, similar to other carbon materials [17,21,22]. After bromination the sample pore volume was also reduced in 10% demonstrating success in bromination. It is interesting to note that the surface area decreases mainly in the micropore area region (Table 3), showing that the bromine molecule is able to diffuse through the porous structure of the Starbon^®^ 700 and react even at the surface of the smaller micropores, which are in larger amount when compared to the mesopores.

The S3 material elemental analysis shows an increase in the carbon content relative to S2, due to the introduction of the bis(oxazoline) ligand rich in carbon (Scheme 1) and the presence of sulfur from the triflate anion coordinated to the copper. From this sulfur content the quantity of copper can be calculated corresponding to 72 µmol/g, which matches well with the quantity of copper directly determined by ICP-OES of 84 µmol/g (Table 1). Although the parent samples S1 and S2 also present some nitrogen, there is also a slight increase in the nitrogen content of the S3 sample, corresponding to 12 µmol/g of the bis(oxazoline) ligand [7,8,9]. Since elemental analysis provides an overall composition of the samples, it may be concluded that S3 contains a large excess of Cu(II) triflate over bis(oxazoline). When compared to S2, a decrease in the bromine content can be seen by XPS (Table 2, Figure 2). A weight loss associated with the peak centered at 215 °C in the derivative of the TG curve can be seen. This all indicates that the reaction between the hydroxyl groups from the CudiolPhBox complex and the reactive bromines introduced at the surface of the Starbon^®^ 700 (Scheme 1) took place. Furthermore, by XPS nitrogen can also be detected confirming the presence of the bis(oxazoline) ligand, as well as copper, fluorine and sulfur from the copper(II) triflate (Table 2). However, in contrast to the elemental and ICP-OES analysis, from the values in Table 3 a higher amount of bis(oxazoline) (98 µmol/g) compared to copper (39 µmol/g) can be calculated. This indicates that there is more chiral ligand at the surface of material than copper. The binding energy for the Cu 3p_3/2_ peak is 933.2 eV and is typical of Cu(II) complexes with bis(oxazoline)-type ligands [23,24] A further decrease in pore volume and surface area can be observed after modification of the surface with the CudiolPhBox complex, also confirming the presence of the complex at the surface of the Starbon^®^ 700 (Table 3).

### 2.2. Catalytic Experiments

**Scheme 3:**
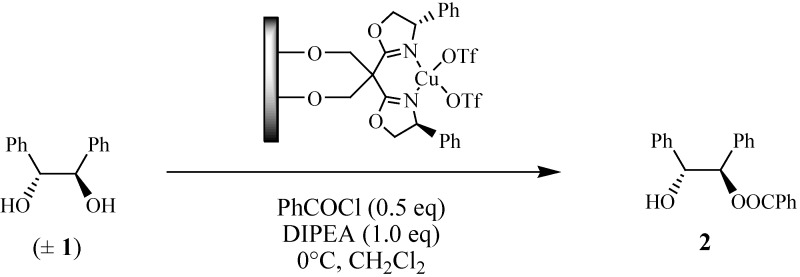
Kinetic resolution of 1,2-diphenylethane-1,2-diol (**3**) with the S3 material.

The copper(II) complexes with bis(oxazoline) ligands act as efficient homogeneous catalysts in several asymmetric organic transformations [1]. In particular, it has been reported that copper(II) complexes with ligand PhBox act as efficient homogeneous catalysts in the kinetic resolution of 1,2-diols [2]. Hence, it was decided to test the S3 material as a heterogeneous catalyst in the asymmetric benzoylation of hydrobenzoin (Scheme 3). The results are compiled in Table 4 together with the homogeneous phase reactions with the reference PhBox and diolPhBox ligands.

**Table 4:** Kinetic resolution of 1,2-diphenylethane-1,2-diol by the S3 material and CuPhBox, CudiolPhBox in homogeneous phase (Scheme 1). ^a^

Catalyst	Cycle	mol ^b^ (%)		%yield ^c^	% *ee* ^d^	*S* ^e^	TON ^f^
		Cu	diolPhBox				
[Cu(OTf)_2_]	1st	0.7		33	0		50
[Cu(OTf)_2_] + PhBox	1st	1.0	1.0	46	84	27	46
[Cu(OTf)_2_] + diolPhBox	1st	1.1	1.1	47	84	26	43
S3	1st	0.7	0.1	38	38	3	55
	2nd	0.7	0.1	20	9	1	30

Notes: **^a^** Reactions performed for 24 h at 0 °C using 0.48 mmol (*R*,*R*)-**3**, 0.48 mmol (*S*,*S*)-**3**, 1.00 mmol DIPEA, 1.0% mol based on Cu and 0.50 mmol of benzoyl chloride in 5.0 mL of CH_2_Cl_2_; **^b^** % of copper and diolPhBox ligand in the catalyst in relation to **1** (see Table 1 and Figure 2); for the recycling experiments corrected for the loss of heterogeneous catalyst weight; **^c^** Isolated yield of **4** (Scheme 3); **^d^** Enantiomeric excess of **4**, determined by HPLC; **^e^** Selectivity (*S*) = ln[1 − yield (1 + ee)]/ln[1 − yield (1 − ee)]; **^f^** TON = moles of isolated **4** /moles of Cu.

The S3 material acts as a heterogeneous catalyst in the kinetic resolution of hydrobenzoin with 38% isolated yield of the monobenzoylated product, in a maximum of 50% yield, and 38% enantioselectivity, confirming the presence of the CudiolPhBox at the surface of the carbonaceous material. Higher catalytic activity was obtained than in all the homogeneous phase reactions. Since the modified CudiolPhBox complex (Scheme 2) behaves as an efficient, enantioselective and selective homogeneous catalyst as the original CuPhBox, the significantly lower enantioselectivity obtained in heterogeneous phase must be due to the low content in the chiral bis(oxazoline) ligand of the S3 material (Table 1). The yield was lower than the homogeneous phase reactions, but slightly higher than the homogeneous phase reaction run with [Cu(OTf)_2_]. The CuPhBox has also been immobilized onto the surface of other mesoporous carbonaceous materials, CMK-3 and SPC, using a different strategy. The monobenzoylated product yield obtained herein is comparable to the one obtained with CMK-3 and higher than the one with SPC as support, but the enantioselectivity is significantly lower. Again this may be attributed to the low content of chiral bis(oxazoline) ligand of the S3 material, whereas the other heterogeneous catalysts present similar quantities of Cu(II) and chiral PhBox essential to obtain good enantioselectivites. The higher copper content obtained by ICP-OES than nitrogen by elemental analysis also suggests that there might be Cu(II) triflate that is directly coordinated to the surface of the Starbon^®^ 700. This would be a non enantioselective way to obtain monobenzoylated product, which may also be contributing to the lowering of the overall S3 heterogeneous catalyst enantioselectivity.

At the end of the reaction with the S3 material, it was removed by vacuum filtration, washed and dried. Then it was used in another cycle of the kinetic resolution of hydrobenzoin, but with reduced monobenzoylated yield and enantioselectivity (Table 4). This may be due to the instability immobilized active species, the copper(II) complex formed with diolPhBox, as found for other immobilized copper(II) bis(oxazoline) systems [5,6,8,9,10].

## 3. Experimental Section

### 3.1. General Information

Copper(II) trifluoromethanosulfonate (copper triflate, [Cu(OTf)_2_], 98%), (*S*)-(−)-2,2'-isopropylidenebis(4-phenyl-2-oxazoline) (**1**, PhBox, 97%), paraformaldehyde (95%), triethylamine (Et_3_N, ≥99%), dry tetrahydrofuran (THF, ≥99.9%), bromine (reagent grade%), and potassium bromide (FT-IR grade, ≥99%) were purchased from Aldrich and used as received. Ethanol (p.a.), methanol (p.a.) were from Riedel de Häen. 1,4-dioxane was from Fisher Scientific and *n*-pentane from VWR. Dichloromethane, acetonitrile, *n*-hexane, isopropanol and ethyl acetate were HPLC grade and from Romil. Elemental analysis and copper ICP-OES were performed in duplicate by CACTI Vigo, University of Vigo (Vigo, Spain). Low resolution ESI-MS was performed at QOPNA of the University of Aveiro (Aveiro, Portugal) and high resolution ESI-MS was performed by CACTI Vigo, University of Vigo. ^1^H- and ^13^C-NMR was performed using a Bruker Avance 300 instrument. FTIR spectra were collected in the range 400–4000 cm^−1^ at room temperature using a resolution of 4 cm^−1^ and 256 scans as KBr pellets with a FT Mattson 7000 galaxy series spectrophotometer; the samples were previously dried in an oven at 100 °C for 6 h.Thermogravimetry analyses (TGA) were performed under nitrogen flow with a ramp of 5 °C/min in a TGA apparatus, model Shimadzu TGA-50. X-ray photoelectron spectroscopy was performed at “Centro de Materiais da Universidade do Porto” (CEMUP, Porto, Portugal), in a KRATOS AXIS Ultra HSA -VISION spectrometer using a nonmonochromatized MgKα radiation (1,253.6 eV). All the materials were compressed into pellets prior to the XPS studies. In order to correct possible deviations caused by electric charge of the samples, the C 1s line at 285.0 eV was taken as internal standard. The elemental contents of the various samples were calculated from the areas of the relevant bands in the high resolution XPS spectra, which were also curve fitted using symmetric Gaussian curves, after fitting to a Shirley background, using XPSpeak version 4.1. Nitrogen adsorption isotherms at −196 °C were measured in an automatic apparatus (Gemini V 2.00 instrument model 2,380; Micromeritics). Before the adsorption experiments the samples were outgassed under vacuum overnight at 120 °C to an ultimate pressure of 1,024 mbar and then cooled to room temperature prior to adsorption.

### 3.2. Synthesis of the Diolphbox

To PhBox (0.0953 g, 0.311 mmol) and paraformaldehyde (0.0254 g, 0.846 mmol, 2.7 eq), dichloromethane (2.5 mL) and 1,4-dioxane (0.6 mL) were added. To this mixture a solution of triethylamine (0.2 mL) in tetrahydrofuran (1.6 mL) was slowly added over a period of 1 h and the solids dissolved gradually during this period. The resulting solution was stirred for 3 days at room temperature and then poured into 8 mL of *n*-pentane. A precipitate appeared immediately which was isolated by vacuum filtration. The solution was evaporated under vacuum and a white foam was obtained (0.052 g, 46% yield). ^1^H-NMR (300 MHz, CDCl_3_), δ/ppm: 7.38–7.25 (m, 10 H), 5.30–5.24 (dd, 2 H, *J* = 10.2 Hz, *J* = 7.9 Hz), 4.74–4.67 (dd, 2 H, *J* = 10.2 Hz, *J* = 8.5 Hz), 4.25–4.15 (m, 6 H). ^13^C-NMR (75 MHz, CDCl_3_), δ/ppm: 166.6, 141.6, 128.8, 127.8, 126.6, 75.0, 69.1, 52.7, 49.9. ESI-HRMS, *m/z*: calculated (C_21_H_23_N_2_O_4_^+^) 367.16523, experimental 367.16575. FTIR, ν/cm^−1^: 2,983 m, 2923 m, 2,853 m (propyl C-H stretching), 1,650 (C=N stretching).

### 3.3. Bromination of the Starbon^®^ 700

To Starbon^®^ 700 (0.5 g), previously dried at 150 °C under vacuum, tetrahydrofuran (THF, 10 mL) and bromine (60 μL) were added. The mixture was stirred at room temperature for 24 h and the red color of the solution faded. The material was isolated by vacuum filtration using 45 μm nylon membranes, washed with THF and dried in an oven at 100 °C under vacuum.

### 3.4. Immobilization of the Cu(II) Complex with Diolphbox Onto the Brominated Starbon^®^ 700

To diolPhBox (0.0279 g, 0.076 mmol) and [Cu(OTf)_2_] (0.0279 g, 0.076 mmol), dichloromethane (60 mL) was added. A blue solution was obtained, but after some minutes stirring it turned green and was left stirring for more 2 h. The brominated Starbon^®^ 700 was added to this solution and refluxed for 36 h. The material was filtered and washed under reflux with fresh dichloromethane (20 mL) in order to remove any physisorbed complex. Finally, the materials were isolated by filtration and dried under vacuum.

### 3.5. Catalysis Experiments

All the catalytic reactions of the prepared materials were performed in batch reactors at atmospheric pressure and with constant stirring. The kinetic resolution of 1,2-diphenylethane-1,2-diol (**1**) was performed at 0 °C using 0.48 mmol (*R*,*R*)-1,2-diphenylethane-1,2-diol, 0.48 mmol (*S*,*S*)-1,2-diphenylethane-1,2-diol, 1.00 mmol DIPEA (170 µL), amount of heterogeneous catalyst containing 0.7% mol Cu and 0.50 mmol of benzoyl chloride (58 µL) in dichloromethane (5.00 mL) [2,9]. The mixture was stirred for 24 h and after filtration of the heterogeneous catalyst the solvent was evaporated from the filtrate and the monobenzoylated product (**2**, Scheme 3) isolated by column chromatography over silica gel using *n*-hexane/ethyl acetate 3:1 as eluent. The **2** enantiomeric excess was determined by HPLC at 254 nm using a Chiralcel OD column (250 mm × 4.6 ID, 5 µm) and *n*-hexane/isopropanol 9:1 as eluent at 1 mL/min. The retention times of the (*R*)-**2** and (*S*)-**2** enantiomers were identified by comparison with those of a racemic **2**. The reaction selectivity (*S*) was calculated based on the isolated yields of **2** and respective enantiomeric excess by using the formulae: ln[1 − yield (1 + ee)]/ln[1 − yield (1 − ee)]. The isolated materials at the end of the reactions were washed extensively with the appropriate solvent, dried under vacuum and reused in another cycle using the same experimental procedure. Control experiments were also performed using the same experimental procedure in homogeneous phase with equimolar quantities of [Cu(OTf)_2_] plus PhBox or diolPhBox in order to compare with the heterogeneous ones.

## 4. Conclusions

A copper(II) complex with a commercial bis(oxazoline) ligand functionalized with CH_2_OH groups was successfully reacted with the brominated surface of a mesoporous carbonaceous material. The material acted as a selective and enantioselective heterogeneous catalyst in the kinetic resolution of hydrobenzoin, with high catalytic activity. Due to the low chiral bis(oxazoline) ligand content the enantioselectivity was reduced in comparison to the homogeneous phase reactions. Upon reuse of the material a further decrease in the product yield and enantioselectivity was observed, probably due to the instability of the copper(II) complex. Further work is being undertaken in order to improve the performance of the immobilized homogeneous catalyst onto porous carbonaceous materials in asymmetric transformations.

## Supplementary Materials

Supplementary File 1
