# Supplementary material for: Synthesis, Immobilization and Catalytic Activity of a Copper(II) Complex with a Chiral Bis(oxazoline)
Source: Molecules. 2014 Aug 11;19(8):11988–98. doi: 10.3390/molecules190811988 (PMC6271255; doi:10.3390/molecules190811988)
Supplement: Supplementary File 1 [file molecules-19-11988-s001.pdf]

Correction of Acknowledgments

In the original published version of this paper we did not correctly and fully acknowledge our financial support. The acknowledgment is hereby additionally published as follows.

This work is financed by FEDER funds through "*Programa Operacional Factores de Competitividade*" – COMPETE and by National funds through FCT – Fundação para a Ciência e Tecnologia under the project CICECO-FCOMP-01-0124-FEDER-037271 (Ref. FCT PEst-C/CTM/LA0011/2013)".
